# Predicting the length of stay at admission for emergency general surgery patients a cohort study

**DOI:** 10.1016/j.amsu.2021.01.011

**Published:** 2021-01-16

**Authors:** T.L. Ward, S.J. Raybould, A. Mondal, J. Lambert, B. Patel

**Affiliations:** a Barts and the London SMD, Dept. Surgery and Surgical Skills, Barts Cancer Institute, London, UK; b St Helen's and Knowsley Teaching Hospitals, Department of Anaesthetics, Prescot, UK; c Homerton University Hospitals Foundation Trust, Department of Surgery, Homerton Row, London, E9 6SR, UK; d Royal London Hospital, Barts Health NHS Trust, Whitechapel Road, London, E1 1FR, UK

**Keywords:** Surgery, Acute care surgery, Emergency general surgery, Healthcare planning, Operational research, Length-of-stay

## Abstract

**Introduction:**

Predicting length of stay (LOS) is beneficial to patients and the health service. When a prolonged LOS is predicted, it gives the opportunity for focused therapies and allocation of resources to reduce this period. In emergency general surgery (EGS) there has been limited investigation of variables that may be important predictors of LOS. This study examines social characteristics alongside measures of severity of acute illness and co-morbidities in an adult EGS population to establish their contribution to LOS.

**Methods:**

Data were collected prospectively from patients at admission including medical variables, demographics, and therapeutic requirements. The length of hospital admission was measured, and multiple regression analysis was used to identify variables which predicted the LOS.

**Results:**

Data were collected from 105 patients. The regression model gave an R^2^ of 0.34, *p* = 0.0006. Barthal index (measure of independence in activities of daily living) was a significant predictor of LOS [logworth 1.649, *p0.02243*]. Housing status and Level of social support both correlated in one-way analysis with LOS.

**Conclusion:**

There are non-surgical variables, measurable at admission which are of significant value in predicting LOS of EGS patients. This warrants further investigation through a larger study to better quantify the contributions of these variables, and establish potential early interventions to reduce the LOS.

## Highlights

• Barthal Index is a significant predictor of length of stay at admission for patients admitted under EGS.
• Unstable housing and a lack of social support independently correlate with a longer length of stay.
• It may be possible to intervene at admission to reduce the length of stay for patients admitted under EGS.

## 1 Introduction

Bed occupancy levels continue to be a major problem within healthcare provision. The National Health Service is under increasing pressure to deliver sustainable patient care and has faced widespread cancelations of elective inpatient care due to high bed occupancy. One of the causes of high bed occupancy is prolonged length of stay (LOS) of patients [1]. LOS has been studied in elective surgical populations and within cohorts with a specific pathology where recovery is partially predicted by the procedure performed [[2], [3], [4]]. Emergency admissions present a challenge in predicting LOS because the procedure and/or recovery required is unknown at admission [5]. LOS in emergency surgical admissions has been studied infrequently in the past, despite having a large impact on bed occupancy.

This study examines potential variables influencing LOS of emergency general surgical (EGS) patients to identify predictive characteristics. Predicting LOS could be used to efficiently deploy staff and allocate resources, aiming to achieve early discharge [6].

Medical variables which determine severity of illness have been studied in other populations, but the applicability to the UK population is limited due to the differences in social care provisions [7]. We postulate that there may be social care characteristics (demographics, housing, social support, and dependence in daily living) as well as medical variables (severity of acute illness and co-morbidities) which predict the LOS of emergency surgical patients.

### 1.1 Aims

The aim of this study is to identify admission characteristics which predict the LOS for emergency surgical patients. The author suggests the following unstudied admission characteristics which may have an impact on the LOS in EGS patients: English language ability, Housing status, Psychiatric medication use, Smoking status, Social support in the community, Barthel Index (measure of independence in activities of daily living) [8].

## 2 Method

Data was collected within 24 h of admission, from patients admitted as emergencies to two London hospitals. Table 1 is the complete list of 24 independent variables which were measured in the data collection.

**Table 1:** Independent variables.

Category	Independent variable
Demographics	Age (Years)
	Sex (M/F)
	Smoking status (Y/N)
	English speaking (Y/N)
Medical	Early Warning Score (EWS)
	Individual Vital signs
	Blood glucose
	ASA score
	Haemoglobin
	White Cell Count
	Platelets
	C-reactive protein
	Serum Creatinine
	eGFR
Therapeutic	Housing status (1 = Stable housing >6months, 2 = stable housing <6months, 3 = Supported housing, 4 = Unstable housing)
	Psychiatric medication use (1 = None, 2 = Antidepressants, 3 = Other psychiatric medication)
	Level of social support (1 = Living with partner, 2 = Sees close friend daily, 3 = Sees close friend < daily, 4 = No support)
	Barthel index (0–100)

English language ability was measured as binary based on the need for an interpreter to answer the questionnaire. Housing status was measured on an ordinal scale of housing stability. Stable housing was defined as privately owned, privately rented or long-term social housing. Supported housing was defined as residential and nursing homes, and sheltered housing. Unstable housing was defined as homelessness, squatting, night-shelters, sofa-surfing etc. Psychiatric medication usage was measured on an ordinal scale (None, Antidepressants, Other psychiatric medications). Social support was measured on an ordinal scale (Living with partner, sees close family/friend daily, sees close family/friend less than daily, Does not have close family/friend in local area) existing packages of care were included within this category. Barthel Index is a numerical scale 0–100 indicating the need for support in activities of daily living (100 = no support required).

### 2.1 Sampling

The sampled population consists of any patient admitted to the hospital for more than 24 h under the emergency general surgical team and not fulfilling the following exclusion criteria:

• Patients under the age of 16 years on the day of admission.
• Patients who were transferred to the care of a non-surgical specialty within 24 h of admission.
• Patients admitted to level 2/3 care within 24 h of admission.

The demographics and medical data are routinely recorded for all patients admitted under an EGS team on the electronic patient record. The therapeutic variables, smoking status and English language status were gained by a patient or close relative interview using a pre-designed questionnaire. The primary outcome (LOS) was collected 30 days later, patients still admitted at 30 days were recorded to have a LOS of 720 h.

One-way analysis was used to identify significant differences for LOS for each independent variable. Multivariate analysis was used to identify correlations between the continuous numeric data. The 10-best performing independent variables were retained for further analysis. Standard least squares regression was used with log transformation of LOS.

Dichotomous variables were compared using student's t-test. Ordinal variables were compared using ANOVA. Continuous variables were plotted with a histogram against length of stay, in 50 h increments. Data and results have been described in-line with the STROCSS 2019 Guideline [9].

### 2.2 Ethics

The study was registered with the research and audit office at each hospital trust, with a senior clinician overseeing the project. The study was registered with IJS Publishing Group ResearchRegistry.com under ID researchregistry6280 [10]. No identifiable patient data was recorded. This was an observational study including patient interviews and electronic data collection, therefore there was no risk of harm to patients. The interview did not impact on patient care.

## 3 Results

Data was collected from 104 patients. The mean values, range and one-way analysis are given in Table 2.

**Table 2:** Descriptive statistics of Dependent and Independent Variables.

Variable	Number of respondents - Mean	Range	One-way analysis
LOS	144.95 h	16 h–769 h	
English speaker	N = 9,Y = 96		t(13.65) = 9.96, *p* = 0.59
Current smoker	N = 70		t(70.48) = -12.14, *p=0.36*
Housing status	1 = 97, 2 = 2,3 = 3, 4 = 4	1:2	D 23.91, p = 0.836
	1 = 133.6 h	1:3	D 197.08, **p=0.039**
	2 = 157.5 h	1:4	D 178.16, **p=0.032**
	3 = 330.7 h	2:3	D 173.17, p = 0.241
	4 = 311.8 h	2:4	D 154.25, p = 0.271
		3:4	D 18.92, p = 0.878
Psych history	AD = 5, N = 96, Y = 4	Y	D 131.81, *p* = 0.081
	Y = 268.6 h	AD	D 67.41, *p* = 0.371
	AD = 204.2 h	N	D 64.40, *p* = 0.535
	N = 136.8 h		
Social support	1 = 41, 2 = 40, 3 = 19, 4 = 5	1:2	D 59.49, *p* = 0.104
	1 = 100.9 h	1:3	D 115.78, ***p*=0.013**
	2 = 160.4 h	1:4	D 32.10, *p* = 0.681
	3 = 216.7 h	2:3	D56.29, *p* = 0.220
	4 = 133.0hrs	2:4	D 27.39, *p* = 0.726
		3:4	D 83.68, *p* = 0.313
Barthal index	95.9	0–100	SD 17.69 SE 1.66
Age	46.59	16–95years	SD 20.22 SE 1.89
Gender	F = 43, M = 62		T(109.49) = 4.98, *p=0.56*
Temperature	36.67	35.0–39.0	SD 0.74 SE 0.07
HR	85.89	51–139	SD 18.47 SE 1.73
SBP	128.97	70–211	SD 24.10 SE 2.26
DBP	79.78	51–105	SD 11.87 SE 1.11
RR	17.4	12–27	SD 2.39 SE 0.22
O2	97.4%	93%–100%	SD 1.70 SE 0.16
EWS	1.17	0–6	
Creatinine	84.79	36–457	SD 46.40 SE 4.36
eGFR	79.96	12->90	SD 16.78 SE 1.58
Hb	132.6	75–169	SD 20.01 SE 1.89
WCC	12.74	2.9–30.7	SD 13.24 SE 1.25
Platelets	276.28	128–755	SD 104.26 SE 9.85
ASA	1.97	1–4	

One-way analysis was carried out for the nominal variables. There was a significantly longer length of stay in patient who reported a housing status of 3 or 4 compared to housing status 1 (1:3 d197.08, *p* = 0.039, 1:4178.16, *p* = 0.032). A significantly longer length of stay was also found in patients who reported a social support status of 3 compared to social support status 1 (1:3 d115.78, *p* = 0.013). There was no significant difference observed within the patients when grouped by use of psychiatric medications.

The continuous variables were plotted on histograms against length of stay (in increments of 50 h). There was a visual association between shorter length of stay and higher Barthal Index. The plot of eGFR also showed an association between a low eGFR and a longer LOS.

Due to non-parametric data and a heavy weighting towards specific variables, transformations were used prior to running regression analysis. Box-cox transformations showed the best lamda to be 0.206, therefore log length of stay was used. The 10 best performing variables were retained in the regression model.

The final 10 variables used in regression were:

• Barthel Index
• Heamoglobin
• Gender
• Social Support
• Psychiatric history
• Respiratory rate
• Housing status
• Age
• English speaking
• eGFR

The regression equation showed an R^2^ of 0.34. With the log values of the predictors as shown in Table 3. The Barthel index showed significant positive association with a shorter length of stay as shown in Table 4.

**Table 3:** Summary of the final regression model.

	R^2^ value	Adjusted R^2^	Root mean square error	Mean of response
Model Summary	0.338829	0.226129	0.817041	4.533435

**Table 4:** The Independent Variables within the regression model.

Independent Variables	Log Worth	*p-Value*
**Barthel Index**	**1.649**	**0.02243**
Hb	1.027	0.09395
Gender	0.934	0.11644
Social Support	0.835	0.14607
Psych History	0.593	0.25537
Respiratory rate	0.569	0.26986
Housing status	0.509	0.30953
Age	0.439	0.36429
English speaker	0.275	0.53061
eGFR	0.256	0.55483

## 4 Discussion

The primary aim of this study was to identify admission characteristics which predict LOS of EGS patients. The hypothesis was that the previously unstudied variables may have an impact of LOS in the EGS population: English language ability, Smoking status, Housing status, Psychiatric medication use, Social support in the community, Barthel Index. Of these variables, Barthel index, eGFR, Social support and Housing status showed correlation with length of stay. Within the regression model Barthel index achieved significance in predicting length of stay.

### 4.1 Descriptive statistics

The average LOS for the cohort was 145 h (6 days). The national average is 4.1 days [9]. The average age of the population was 46.6 years, which is younger than the national average 55.8 year for general surgery patients overall [11]. There may be several factors contributing to these discrepancies. Firstly, the population sampled was taken from two London hospitals. The different boroughs of London have varying average ages, levels of deprivations and endemic diseases. The exclusion criteria for this study excluded patients transferred to level 2/3 care within 24 h of their admission, which may have contributed to the difference in the demographics against the national averages.

### 4.2 Exclusion criteria

The exclusion criteria for under 16 years on day of admission and patients transferred to a non-surgical specialty were aimed at focusing the population to those cared for by the emergency general surgical clinicians, nursing teams and allied specialties. These criteria will vary in each hospital, as there is heterogeneity in policy across different sites.

The exclusion of patients admitted to level 2/3 care was to exclude extreme outliers in LOS. There are already multiple studies which examine LOS for patients undergoing an admission to ITU [13]. These studies use variables specific to the physiological changes and associated morbidity with an ITU admission. This exclusion criteria have likely resulted in a less acutely unwell population being demonstrated in this study, as can be seen by the ranges of the vital signs and biochemical markers in some of the independent variables.

### 4.3 Initial analysis

The initial one-way analysis of the independent variables showed that both housing status and social support correlate to LOS. Patients with housing status 1 (lived in their own home/rented property for >6 months) stayed in hospital for a significantly shorter time than patients with housing status 3 (unstable housing) or 4 (homeless). The frequency of housing status 2–4 was very low in the cohort, which may have reduced the impact this had in the regression model. Although the data was treated as nominal in analysis, the assumption was made that the housing status values were likely to be ordinal in their impact on LOS. This was not shown in the mean values, as the mean LOS was shorter in housing status 4 vs 3. This may be related to patient specific factors which determine the location of discharge; patients who are homeless may wait less time for placement in hostels than older patients who own their own home but await medical equipment, or alternative residential/nursing placement.

Social support showed a more heterogenous cohort. There was a significantly longer LOS in patients who reported social support status of 3 vs 1. This may be explained by the necessity for delayed discharge in patients who have less social support in the community. These patients may require longer to recover in hospital or initiation of a package of care at home, prior to discharge. However, there was no statistical significance seen between patients with social support level 1 and 4. This may be due to the low numbers of respondents who self-report as social status 4 (having no close relative to provide care), or specific characteristics of these patients, but further research is required to explore this.

LOS showed visual correlation with eGFR in the histogram analysis, which probably represents a more co-morbid patient, who could be expected to require a longer stay in hospital, as demonstrated in other studies [7,14].

### 4.4 The regression model

Within the regression model a low *Barthel index* was found to be a significant predictor of length of stay. The Barthel index is a measure of the patient's independence in performing their activities of daily living. The index uses a numerical scale from 0 to 100 measuring parameters of continence, feeding, grooming, hygiene, mobility and transferring. A score of 100 denotes a person requiring no assistance. These parameters need to be accounted for to achieving safe discharge, therefore it follows that greater dependence will lead to a longer length of stay. If these parameters can be addressed at admission it may be possible to reduce LOS.

There was minimal correlation between LOS and surrogate markers for severity of disease (basic observations and biochemical markers). eGFR showed possible correlation in the histogram analysis, but was found to perform poorly as a predictor of LOS in the regression model. This may be accounted for by the exclusion criteria as very unwell patients were likely excluded as they may have been transferred to a higher level of care within 24 h of their admission. Haemoglobin was the second strongest predictor within the regression model and thus may have some value in predicting the LOS but did not achieve significance.

The R^2^showed that this model accounts for 34% of the variance in LOS in the cohort which is to be expected with a data set taken at admission. This cohort included patients discharge without undergoing an operation and those who had operative interventions and required a period of rehabilitation post-operatively. The factors which would have influenced this are likely to include radiological diagnoses, and clinical decision making, and operative intervention and complications, which are beyond the scope of this study. What this study demonstrates is that there may be non-surgical predictors of LOS which can be identified at admission. This means that early therapeutic interventions may have a role in reducing prolonged LOS from the day of admission. This is likely to include physiotherapy, occupational therapy, and social services support to optimise function and facilitate safe discharge. It may be possible to allocate resources to initiate these interventions earlier in the hospital stay e.g. on the day of admission, to facilitate earlier discharge.

This study was limited to two hospitals based in two of London's boroughs. The cohort studied may not represent patients across the country and furthermore, the resources available to facilitate discharge will vary depending on geographical location and the local authority.

## 5 Conclusion

This study has demonstrated that there are variables measurable at admission which correlate with a LOS, and which may predict the LOS in patients admitted as emergencies under general surgical teams. Further investigation is needed to better quantify the impact that each of these variables has independently, so that they can be utilised in resource allocation and planning of EGS services. Following this, it may be possible to assess the impact of focused intervention at admission in achieving earlier discharge.

## Sources of funding for your research

No funding was received for this research.

## Ethical Approval

Registered as an audit in each participating hospital site.

## Research Registration Unique Identifying Number (UIN)

1. Name of the registry: Researchregistry.com.

2. Unique Identifying number or registration ID: researchregistry6280.

3. Hyperlink to your specific registration (must be publicly accessible and will be checked): https://www.researchregistry.com/browse-the-registry#home/registrationdetails/5fbbde7f4c5355001c9d1df4/.

## Author contribution

Mr. T.L. Ward: Conceptualization, Data curation, Formal analysis, Methodology, Writing-original draft.

Dr. R.J. Raybould: Formal analysis, Writing-review and editing.

Dr. A. Mondal: Data curation.

Dr. J. Lambert: Data curation.

Mr. B. Patel: Supervision

## Guarantor

Corresponding Author: Mr. Thomas Lee WARD, Barts and the London SMD, Dept. Surgery and Surgical Skills, Barts Cancer Institute.

Email: tom.ward3@nhs.nettomwardmsm@hotmail.com.

Address: Flat 5, 31-33 Ivanhoe Road, Liverpool, L17 8XF, United Kingdom.

## Provenance and peer review

Not commissioned, externally peer-reviewed.

## CRediT authorship contribution statement

**T.L. Ward:** Conceptualization, Data curation, Formal analysis, Methodology, Writing - original draft. **S.J. Raybould:** Formal analysis, Writing - review & editing. **A. Mondal:** Data curation. **J. Lambert:** Data curation. **B. Patel:** Supervision.

## Declaration of competing interest

None.

## References

[1] Lewis R., Edwards N. (2015). Research report: improving length of stay: what can hospitals do?. Nuffield Trust.

[2] Collins T.C., Daley J., Henderson W.H., Khuri S.F. (1999). Risk factors for prolonged length of stay after major elective surgery. Ann. Surg..

[3] Handoll H.H., Cameron I.D., Mak J.C., Finnegan T.P. (2009). Multidisciplinary rehabilitation for older people with hip fractures. Cochrane Database Syst. Rev..

[4] Handoll H.H., Sherrington C., Mak J.C. (2011). Interventions for improving mobility after hip fracture surgery in adults. Cochrane Database Syst. Rev..

[5] Watson R., Crump H., Imison C., Currie C., Gaskins M. (2016). Emergency General Surgery: Challenges and Opportunities.

[6] Fox M.T., Persaud M., Maimets I., Brooks D., O'Brien K., Tregunno D. (2013). Effectiveness of early discharge planning in acutely ill or injured hospitalized older adults: a systematic review and meta-analysis. BMC Geriatr..

[7] Chuang M.T., Hu Y.H., Lo C.L. (2018). Predicting the prolonged length of stay of general surgery patients: a supervised learning approach. Int. Trans. Oper. Res..

[8] Mahoney F.I., Barthel D.W. (1965). Functional evaluation: the Barthel Index: a simple index of independence useful in scoring improvement in the rehabilitation of the chronically ill. Md. State Med. J..

[9] Agha R., Abdall-Razak A., Crossley E., Dowlut N., Iosifidis C., Mathew G., for the Strocss Group (2019). The STROCSS 2019 guideline: strengthening the reporting of cohort studies in surgery. Int. J. Surg..

[10] Ward ThomasLee Browse the registry - researchregistry6280. ResearchRegistry.com. https://www.researchregistry.com/browse-the-registry#home/registrationdetails/5fbbde7f4c5355001c9d1df4/.

[11] NHS Digital (2017). Hospital Admitted Patient Care Activity 2016-2017.

[13] Goldfarb M.J., Bibas L., Bartlett V., Jones H., Khan N. (2017). Outcomes of patient-and family-centered care interventions in the ICU: a systematic review and meta-analysis. Crit. Care Med..

[14] Liangos O., Wald R., O'Bell J.W., Price L., Pereira B.J., Jaber B.L. (2006). Epidemiology and outcomes of acute renal failure in hospitalized patients: a national survey. Clin. J. Am. Soc. Nephrol..

